# Circadian rhythm pattern of symptom onset in patients with ST-segment elevation myocardial infarction in the Chinese population

**DOI:** 10.3389/fcvm.2024.1393390

**Published:** 2024-12-10

**Authors:** Yibo Guo, Lina Cui, Lulu Li, Zhuozhong Wang, Chao Fang, Bo Yu

**Affiliations:** ^1^ Department of Cardiology, The 2nd Affiliated Hospital of Harbin Medical University, Harbin, China; ^2^ State Key Laboratory of Frigid Zone Cardiovascular Diseases (SKLFZCD), Harbin Medical University, Harbin, China; ^3^ The Key Laboratory of Myocardial Ischemia, Chinese Ministry of Education, Harbin, China

**Keywords:** ST-segment elevation myocardial infarction, circadian rhythm, bimodal, outcomes, primary peak

## Abstract

**Background:**

The peak incidence of cardiovascular diseases (CVD) usually occurs in the morning. This study aimed to investigate the exact distribution pattern of peak incidence of ST-segment elevation myocardial infarction (STEMI) in the Chinese population, and to explore whether it is associated with the prognosis.

**Methods:**

This study included 7,805 patients with STEMI from the multicenter, prospective AMI cohort in China, for whom had a definite time of symptom onset. In the overall population and the predefined subgroup populations, the circadian rhythms of STEMI onset were statistically analyzed. Then patients were divided into four groups based on the time of onset (6 h interval) to assess the association of symptom onset time and major adverse cardiovascular and cerebrovascular events (MACCE) after discharge.

**Results:**

The onset of STEMI had a bimodal distribution: a well-defined primary peak at 8:38 AM [95% confidence interval (CI): 7:49 to 9:28 AM], and a less well-defined secondary peak at 12:55 PM (95% CI: 7:39 AM to 18:10 PM) (bimodal: *P* < 0.001). A similar bimodal circadian rhythm pattern was observed in subgroups of patients with STEMI defined with respect to day of the week, age, sex, and coronary risk factors. Notedly, the two peaks on Sunday were significantly later than other days, and the secondary peaks became clear and concentrated. In addition, no significant difference was found in MACCE among the four groups (*P* = 0.905).

**Conclusions:**

In the Chinese population, the onset of STEMI exhibited a bimodal circadian rhythm pattern, with a clear primary peak and a less clear secondary peak. One-year clinical outcomes were unrelated to the timing of STEMI onset.

## 1 Introduction

Cardiovascular disease (CVD) is the primary cause of premature death and disability and the leading global burden of disease today (1). The majority of CVDs, including myocardial infarction, stroke, arrhythmia, and sudden cardiac death, have been found to have a distinct circadian rhythm, with the incidence generally peaking in the morning (6:00–12:00 AM) (2–6). This is related to periodic changes in sympathetic tone, blood pressure, endothelial function, platelet aggregation and plasma fibrinolytic activity during morning rise (7–9). A small number of studies additionally suggested that the afternoon or evening might be the second peak of CVD onset (6, 10, 11). Whether this bimodal distribution of rhythms is also present in Chinese STEMI patients remains unclear. The impact of STEMI onset time on the future adverse cardiac events is controversial. Some studies showed a worse prognosis for patients with night or early morning onset, while others showed that the onset time of STEMI is not associated with adverse events (3, 12–14). Therefore, the present study aimed to assess the circadian rhythm pattern of STEMI onset in the Chinese population, and to compare the impact of different onset times on the clinical outcomes of STEMI patients receiving standardized treatment.

## 2 Methods

### 2.1 Study design and participants

Patients were recruited from a multi-center, prospective, observational cohort study of 12,043 patients with AMI hospitalized at 20 tertiary hospitals from China between December 2017 and December 2019 (NCT03297164). Of those patients, 4,229 cases were excluded for the following reasons: patients with non-ST-segment elevation acute myocardial infarction (NSTEMI) who rarely present with typical chest pain (*n* = 3,609), patients with STEMI who have an uncertain time of onset of chest pain (*n* = 629). Finally, 7,805 patients with STEMI with a clear time of onset were included in the present study.

**Figure 1:**
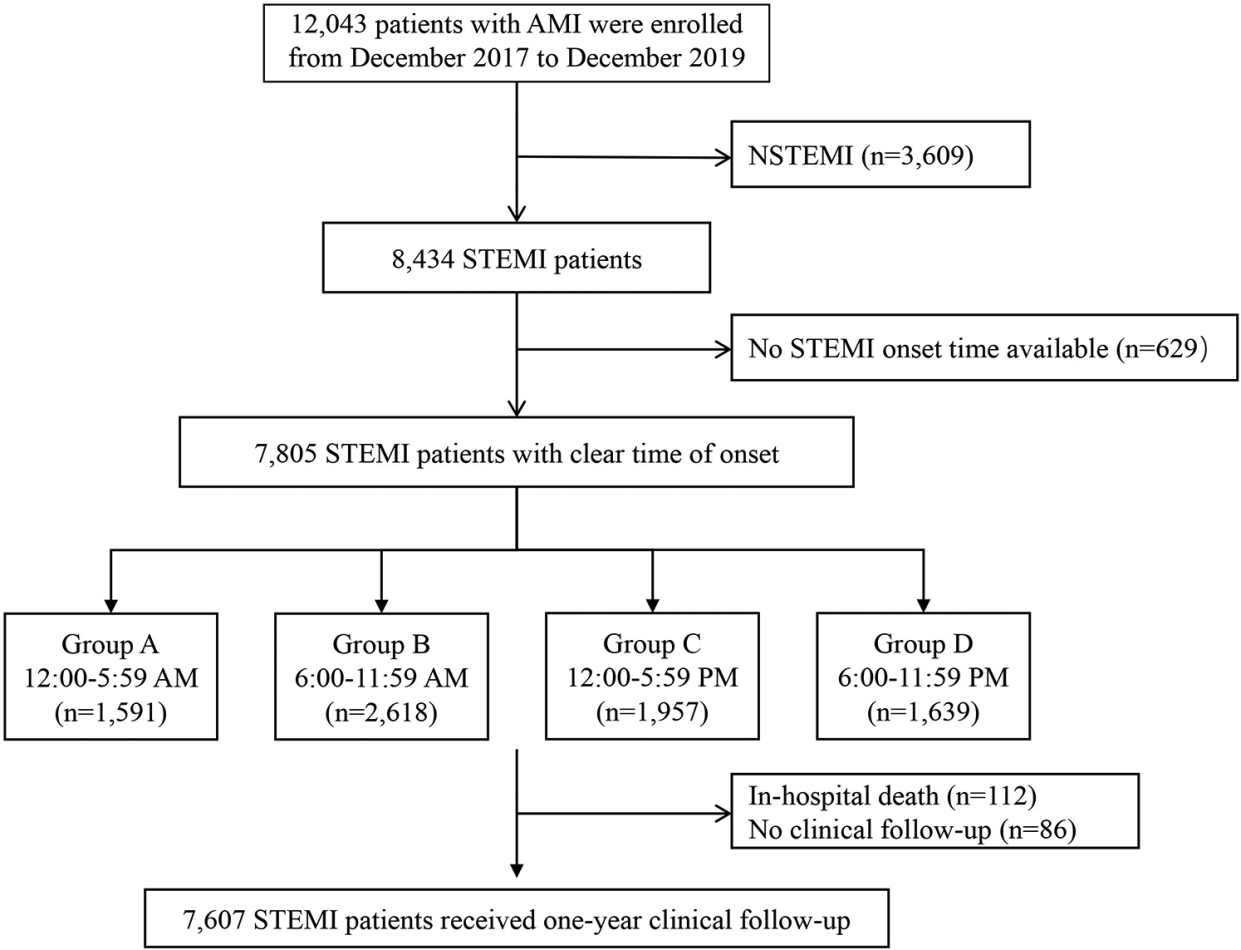
Study flow chart. AMI, acute myocardial infarction; NSTEMI, ST-segment elevation myocardial infarction; STEMI, ST-segment elevation myocardial infarction.

STEMI onset time was defined as the time of occurrence of the patient's perceived persistent chest pain, which was obtained from patients or their guardians. All enrolled patients were further divided into four groups according to the time of STEMI onset (6 h interval) based on previous reports (3): Group A, 12:00 AM-5:59 AM (*n* = 1,591); Group B, 6:00 AM-11:59 AM (*n* = 2,618); Group C, 12:00 PM-5:59 PM (*n* = 1,957); Group D, 6:00 PM-11:59 PM (*n* = 1,591). The study flow chart is shown in Figure 1.

AMI was diagnosed according to the Fourth Universal Definition of Myocardial Infarction, including STEMI and NSTEMI (15). STEMI was defined as continuous chest pain that lasted > 30 min, arrival at the hospital within 12 h of the symptom onset, ST-segment elevation > 0.1 mV in at least 2 contiguous leads, or new-onset left bundle-branch block on the 12-lead electrocardiogram (ECG), and elevated of cardiac markers (creatine kinase-myocardial band or troponin T/I) (16). NSTEMI was defined as ischemic symptoms in the absence of ST-segment elevation on the ECG, with elevated cardiac markers levels (17). Definitions of traditional coronary risk factors are included in the Supplementary Material.

The study was approved by the Ethics Committee of The Second Affiliated Hospital of Harbin Medical University (Harbin, China), complied with the ethical guidelines of the Declaration of Helsinki. All patients provided written informed consent before the start of the study.

### 2.2 Endpoints and clinical follow-up

Patients received scheduled follow-up at 1, 6, and 12 months after discharge via clinical visit or telephone interview. The primary endpoint was major adverse cardiovascular and cerebrovascular events (MACCE) within one year after discharge, which were defined as the composite of all-cause death, nonfatal recurrent myocardial infarction, coronary revascularization, and nonfatal stroke. Detailed definitions of individual events are provided in the Supplementary Material.

### 2.3 Statistical analysis

All statistical analysis was performed using SAS V.9.4 (SAS Institute, Inc, Cary, North Carolina) and R V.4.1.2 (R Foundation for Statistical Computing, Vienna, Austria). A two-tailed *P*-value < 0.05 indicates that the difference is statistically significant. The nonparametric one-sample Kolmogorov–Smirnov test was used to assess the normality of continuous variables. Normally distributed variables are described as mean ± standard deviation (SD) and compared by using the Analysis of Variance (ANOVA), whereas non-normally distributed variables are described as median [interquartile range (IQR)] and compared by using the Kruskal Wallis *H* test. Categorical variables were presented as number (percentage), and compared using either a Chi-square test or Fisher exact test, as appropriate. Multivariable Cox proportional hazards model was performed to evaluate the association between onset time and clinical outcomes. The results are reported as hazard ratio (HR) and 95% CI, using Group B as reference.

For the periodic onset density of STEMI at different time periods, the optimal parameters fit the von Mises distribution using maximum likelihood estimation, and peak shape was analyzed using the Kolmogorov-Smirnov test to assess whether the circadian pattern of STEMI onset had uniform (no peak), unimodal (one peak) or bimodal distribution (two peaks). We performed stratified analysis by day of the week, age, sex, and coronary risk factors, evaluated the heterogeneity in circadian rhythm distribution between subgroups.

## 3 Results

### 3.1 Baseline characteristics

From December 2017 to December 2019, a total of 7,805 patients with STEMI were included in the final analysis. The median age of patients was 61.7 years, 5,574 (74%) were male, and 2,618 (33.4%) presented with symptoms between 12:00 AM and 5:59 AM. The baseline clinical characteristics of patients in different groups are summarized in Table 1. Compared to the other three time periods, patients in Group A were more likely to be older (*P* < 0.001), and had a longer time from symptom onset to hospital admission (*P* < 0.001). There were no statistically significant differences in receiving revascularization therapy among the four groups (*P* > 0.05).

**Table 1:** Baseline characteristics among groups based on time of STEMI onset.

	Group A (*n* = 1,591)	Group B (*n* = 2,618)	Group C (*n* = 1,957)	Group D (*n* = 1,639)	*P* value
Demographics					
Age, years	62.6 (53.9–69.7)	62.2 (53.4–69.5)	61.8 (53.5–68.9)	60.3 (51.9–68.0)	**<0**.**001**
Male, *n* (%)	1,156 (72.7)	1,918 (73.3)	1,450 (74.1)	1,250 (76.3)	0.086
BMI, kg/m^2^	24.6 (22.8–27.0)	24.5 (22.5–26.7)	24.8 (22.9–27.0)	24.7 (22.8–27.0)	0.050
Urban residence, *n* (%)	1,220 (76.7)	1,939 (74.1)	1,478 (75.5)	1,263 (77.1)	0.100
Risk factors, *n* (%)					
Dyslipidemia	42 (2.6)	84 (3.2)	58 (3.0)	57 (3.5)	0.546
Hypertension	787 (49.5)	1,238 (47.3)	964 (49.3)	765 (46.7)	0.234
Diabetes	370 (23.3)	584 (22.3)	437 (22.3)	372 (22.7)	0.894
Obesity	53 (3.3)	70 (2.7)	51 (2.6)	48 (2.9)	0.555
Smokers	686 (43.1)	1,050 (40.1)	855 (43.7)	774 (47.2)	**<0**.**001**
Drinkers	326 (20.5)	502 (19.2)	378 (19.3)	345 (21.1)	0.393
Previous history, *n* (%)					
Previous PCI	75 (4.7)	147 (5.6)	100 (5.1)	87 (5.3)	0.635
Previous CABG	3 (0.2)	12 (0.5)	3 (0.2)	4 (0.2)	0.259
Previous stroke	209 (13.1)	349 (13.3)	259 (13.2)	187 (11.4)	0.267
Onset-to-admission time, min	342.0 (208.0–606.0)	281.5 (180.0–471.5)	266.5 (168.0–419.5)	255.5 (158.0–570.0)	**<0**.**001**
Laboratory data on admission					
TC, mg/dl	175.6 (150.4–203.4)	176.7 (151.2–206.1)	177.1 (152.7–206.1)	176.3 (150.8–203.8)	0.412
TG, mg/dl	128.4 (92.1–186.0)	131.1 (95.7–187.8)	126.7 (88.6–184.2)	127.5 (85.9–186.9)	**0**.**012**
LDL-C, mg/dl	104.0 (77.0–128.8)	104.0 (78.9–132.6)	106.3 (81.2–132.3)	105.6 (78.5–131.5)	**0**.**042**
HDL-C, mg/dl	43.3 (36.7–54.5)	43.3 (36.0–54.9)	44.1 (36.3–53.8)	43.3 (36.7–54.1)	0.910
Hs-CRP, mg/L	4.8 (1.9–11.7)	5.0 (2.0–11.4)	4.8 (2.0–10.9)	4.2 (1.8–10.8)	0.202
CK-MB, U/L	17.2 (3.0–75.1)	12.4 (2.5–59.2)	11.9 (2.9–60.5)	11.4 (2.5–61.3)	0.050
cTnI, μg/L	3.2 (0.4–19.6)	2.5 (0.3,15.9)	2.0 (0.2–14.6)	2.3 (0.3–15.4)	**0**.**023**
NT-pro BNP, pg/ml	655.0 (190.0–1777.0)	669.0 (183.0–1659.0)	575.2 (168.0–1516.0)	575.0 (146.0–1589.0)	0.050
FBG, mmol/L	6.4 (5.3–8.3)	6.2 (5.2–8.1)	6.63 (5.5–8.7)	6.8 (5.5–9.1)	**<0**.**001**
HbA1c,%	5.9 (5.6–7.1)	6.0 (5.6–7.2)	6.0 (5.6–7.1)	5.9 (5.6–7.1)	0.062
LVEDD, mm	48.0 (45.0–51.0)	48.0 (44.0–51.0)	47.0 (44.0–51.0)	47.3 (44.7–51.0)	0.250
LVEF, %	56.8 (49.0–61.0)	57.0 (50.0–61.0)	57.0 (49.0–61.0)	56.8 (49.0–61.0)	0.356
Treatment, *n* (%)					
Reperfusion	1,479 (93.0)	2,431 (92.9)	1,843 (94.2)	1,526 (93.2)	0.316
Thrombolysis	60 (3.8)	89 (3.4)	69 (3.5)	47 (2.9)	0.538
PCI	1,466 (92.1)	2,409 (92.0)	1,828 (93.4)	1,516 (92.6)	0.320
Thrombus aspiration	545 (41.4)	896 (42.2)	718 (43.9)	582 (43.1)	0.542
Stent implantation	1,180 (74.2)	1,964 (75.0)	1,506 (77.0)	1,231 (75.1)	0.249
In-hospital death	24 (1.51)	40 (1.53)	27 (1.38)	21 (1.28)	0.911

Values are presented as *n* (%) or median (interquartile range). A *P* value of <0.05 was considered statistical significance, shown in bold. BMI, body mass index; CABG, coronary artery bypass grafting; CK-MB, creatine kinase-myocardial band; cTnI, cardiac troponin I; FBG, fasting blood-glucose; HDL-C, high-density lipoprotein cholesterol; Hs-CRP, high sensitivity C reactive protein; HbA1c, hemoglobin A1C; LDL-C, low-density lipoprotein cholesterol; LVEDD, left ventricular end diastolic diameter; LVEF, left ventricular ejection fraction; NT-pro BNP, N-terminal pro–brain natriuretic peptide; PCI, percutaneous coronary intervention; TC, total cholesterol; TG, triglyceride.

### 3.2 Bimodal circadian rhythm pattern of STEMI onset

**Figure 2:**
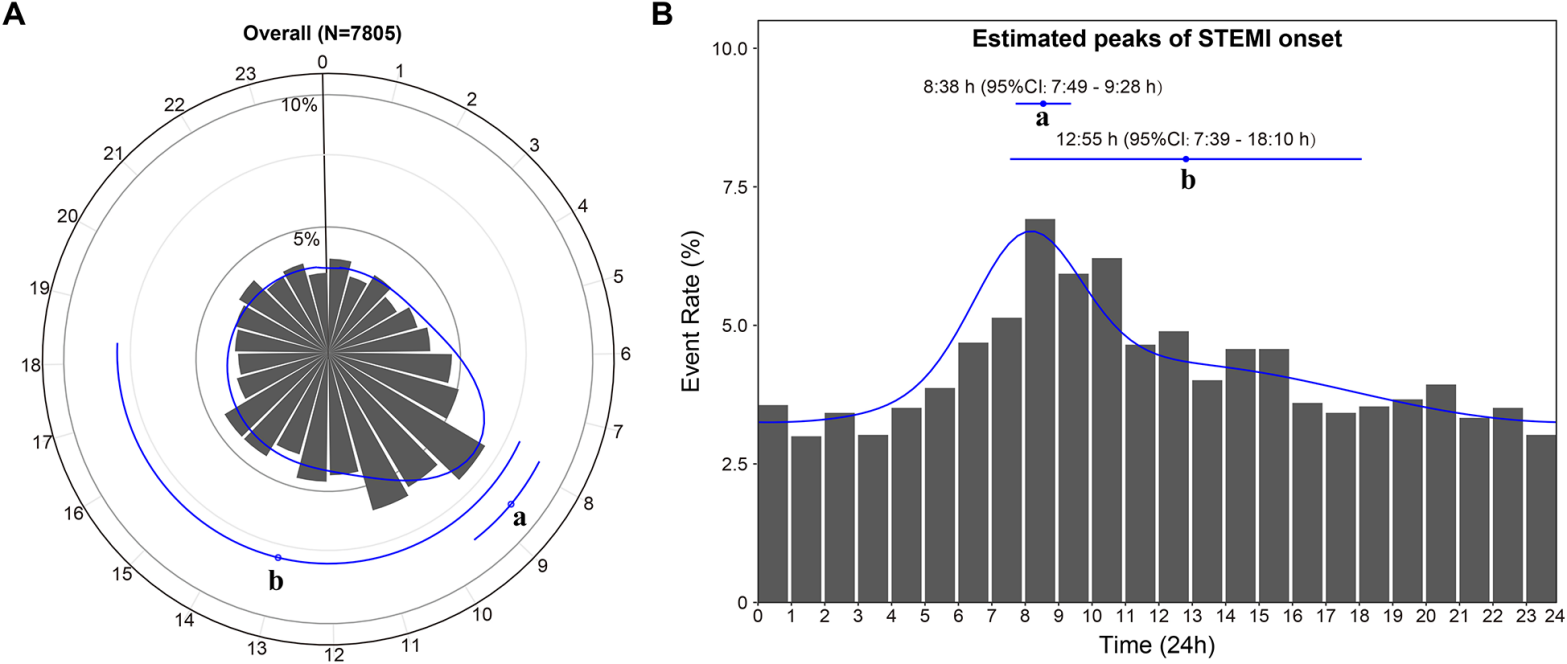
Circadian rhythm pattern of STEMI onset in the overall study cohort. The primary peak **(a)** and secondary peak **(b)** of the onset of STEMI within 24 h are depicted in the circle plot **(A)** and histogram **(B)**. The estimated peak onset times and 95% CIs are represented by the dots with error lines, while the solid line corresponds to the fitted von Mises distribution. CI, confidence interval; STEMI, ST-segment elevation myocardial infarction.

Figure 2 shows the best-fit curve for the temporal distribution of STEMI onset. In the overall study cohort, the onset of STEMI exhibited a bimodal distribution over 24 h, with a significant primary peak at 8:38 AM [95% confidence interval (CI): 7:49 to 9:28 AM] and a small second peak at 12:55 PM (95% CI: 7:39 AM to 18:10 PM) (bimodal: *P* < 0.001).

### 3.3 Circadian rhythm variations of the day of the week and the quarters of the year

**Figure 3:**
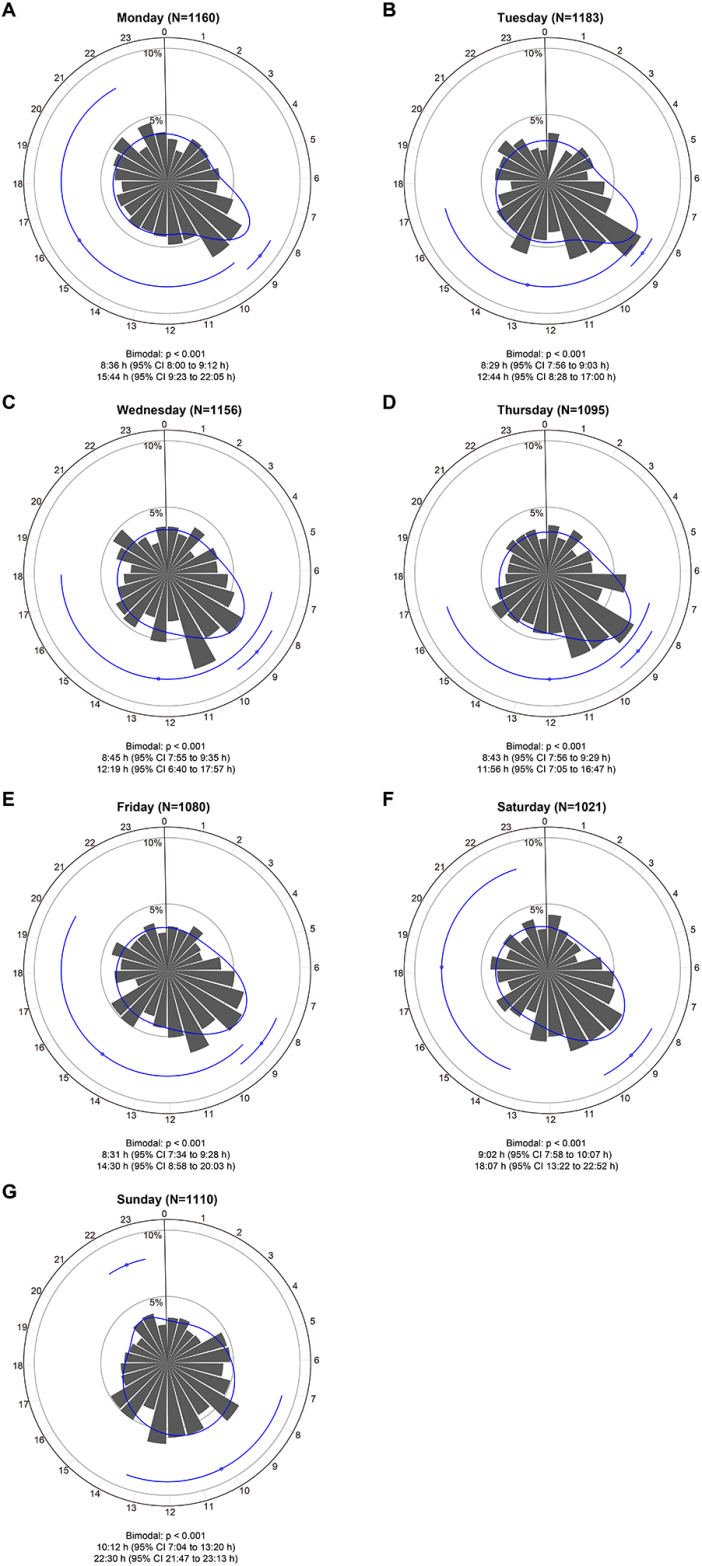
Circadian rhythm pattern of STEMI onset based on the day of the week. Panel **(A–G)** shows circular plots fitting the von Mise distribution based on the day of the week. The estimated peak onset times and 95% CIs are shown below each circular plot. *P* values are used to assess whether the circadian rhythm pattern of STEMI onset was uniform, unimodal or bimodal. CI, confidence interval; STEMI, ST-segment elevation myocardial infarction.

The onset of STEMI still had two peaks on each day of the week (bimodal, all *P* < 0.001), with timing varying depending on the day (Figure 3). On weekdays, the time of the first peak remained consistent, at around 8:30 AM. And the morning peak became sharper on Monday and Tuesday (Figures 3A,B). Compared with other dates, the Sunday delayed the occurrence times of first peak [10:12 AM (95% CI: 7:04 AM to 13:20 PM)] and second peak [22:30 PM (95% CI: 21:47 to 23:13 PM)], and exhibited a highly concentrated evening peak time **(**Figure 3G). Interestingly, there is still a bimodal distribution of STEMI onset in quarter 1 (January-March), quarter 2 (April-June), and quarter 3 (July-September) (bimodal, all *P* < 0.001) (Supplementary Figures S1A–C), whereas the unimodal distribution of STEMI onset can be found in quarter 4, with only one morning peak at 8:59 AM (bimodal: *P* > 0.05) (Supplementary Figure S1D).

### 3.4 Bimodal circadian rhythms in different subgroups

**Figure 4:**
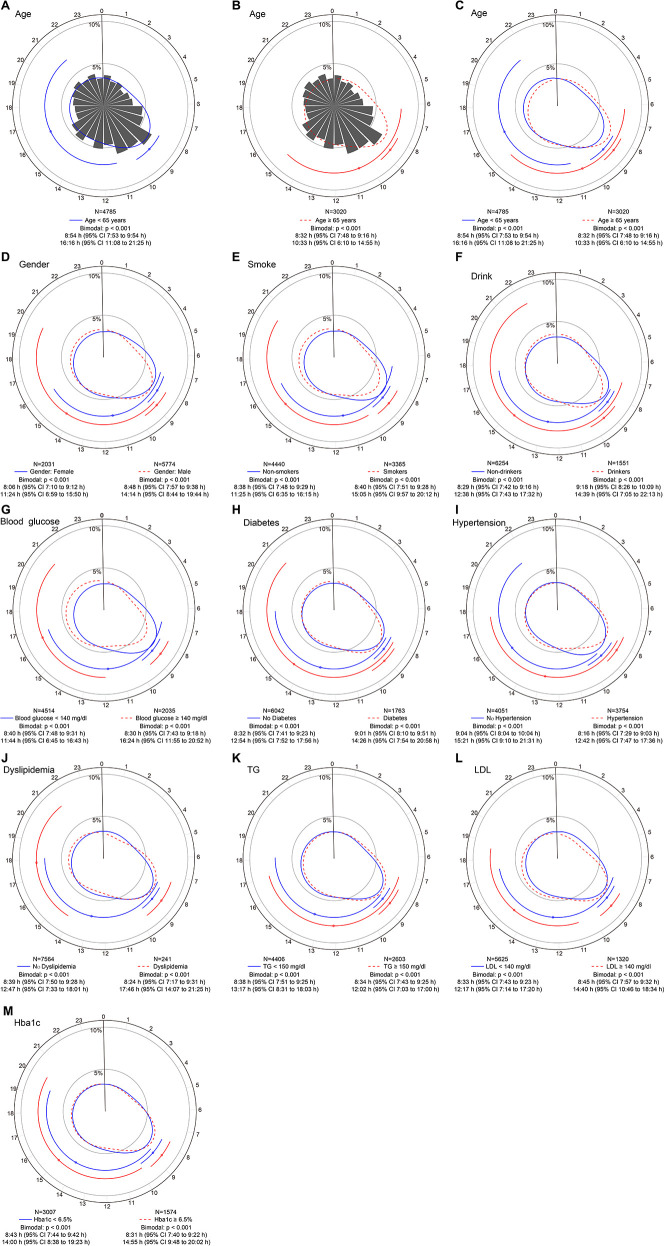
Circadian rhythm pattern of STEMI onset based on baseline factors. Panel **(A–C)** shows circular plots fitting the von Mise distribution in subgroups aged ≥65 years and <65 years. Panel **(D–M)** show circular plots fitting the von Mise distribution in subgroups stratified by sex, smoking habits, drinking habits, blood glucose levels, diabetes, hypertension, dyslipidemia, triglyceride (TG) levels, low-density lipoprotein (LDL) levels and glycated hemoglobin (HbA1c) levels. The estimated peak onset times and 95% CIs are shown below each circular plot. *p* values are used to determine whether the circadian rhythm pattern of STEMI onset was uniform, unimodal or bimodal. CI, confidence interval; STEMI, ST-segment elevation myocardial infarction.

Each subgroup's STEMI distribution still showed a similar bimodal pattern, with the morning peaks showing relatively fixed time and being much clearer (all *P* < 0.001) (Figure 4). We noted some differences in the secondary peaks among the three STEMI subgroups. The second peak's time was shifted forward and close to the primary peak in STEMI patients aged ≥ 65 years (Figure 4B). Both peaks tend to occur in the morning (primary peak at 8:32 AM and secondary peak at 10:33 AM). There was no difference between the male and the female. In patients with dyslipidemia or blood glucose ≥ 140 mg/dl, the second peak of STEMI onset both appeared somewhat late (Figures 4G,J).

### 3.5 Clinical outcomes

The median follow-up of 1-year was completed in 97.5% (7,607/7,805) of patients. There were 112 patients (1.4%) who died prior to discharge, and there was no significant difference in in-hospital mortality among the four groups (*P* = 0.911) (Table 1). There was no significant difference in the incidence of periprocedural complications (all *P* > 0.05) (Supplementary Table S1) and bleeding during hospitalization (*P* > 0.05) (Supplementary Table S2) among the four groups. The medication at discharge was also comparable (all *P* > 0.05) (Supplementary Table S3). No difference was also observed in angiographic and PCI-related results (Supplementary Table S4).

During the one-year follow-up period, 400 patients (5.2%) experienced MACCE after discharge, including 186 deaths (2.4%), 45 recurrent myocardial infarctions (0.65%), 169 revascularizations (2.1%), and 52 strokes (0.5%). There were no significant differences in the primary endpoint at one year among the four groups (*P* = 0.905) (Table 2). Multivariate Cox regression models (Supplementary Table S5) showed that the independent predictors of 1-year MACE were age (HR: 1.03; 95% CI: 1.02–1.05) and cardiogenic shock at admission (HR: 129.78; 95% CI: 22.45–613.66). Age (HR: 1.04; 95% CI: 1.01–1.07), cardiogenic shock at admission (HR: 57.99; 95% CI: 5.66–594.12), and the femoral approach (HR: 1.98; 95% CI: 1.13–3.49, radial approach as reference) can independently predict all-cause death. The timing of STEMI onset still cannot independently predict MACCE and all-cause death after adjustment for these variables. In addition, when we conducted the analysis only with patients admitted before 2019, the result was similar to those in the main analyses **(**Supplementary Table S6**)**.

**Table 2:** Clinical outcomes at one year according to time of STEMI onset.

Events	Group A (*n* = 1,550)	Group B (*n* = 2,549)	Group C (*n* = 1,910)	Group D (*n* = 1,598)	*P* value
MACCE, *n* (%)	81 (5.2)	128 (5.0)	105 (5.4)	86 (5.3)	0.905
All-cause death, *n* (%)	41 (2.6)	53 (2.1)	55 (2.9)	37 (2.3)	0.343
Recurrent MI, *n* (%)	3 (0.2)	16 (0.6)	17 (0.9)	9 (0.6)	0.067
Revascularization, *n* (%)	31 (2.0)	58 (2.3)	35 (1.8)	36 (2.2)	0.733
Stroke, *n* (%)	9 (0.6)	15 (0.6)	15 (0.8)	13 (0.8)	0.737
Bleeding	24 (1.5)	56 (2.2)	31 (1.6)	33 (2.0)	0.356
Intracranial hemorrhage	1 (0.1)	2 (0.1)	2 (0.1)	4 (0.2)	0.496
Gastrointestinal bleeding	5 (0.3)	15 (0.6)	8 (0.4)	6 (0.4)	0.593
Other bleeding	18 (1.1)	39 (1.5)	21 (1.1)	23 (1.4)	0.568

Data are presented as number (%). MACCE was defined as the composite of all-cause death, recurrent myocardial infarction, revascularization, and stroke. MACCE, major adverse cardiovascular and cerebrovascular events; MI, myocardial infarction; STEMI, ST-segment elevation myocardial infarction.

## 4 Discussion

In this large multi-center, prospective study, circadian rhythm variation in symptom onset was assessed in 7,805 hospitalized STEMI patients, expanding previous clinical understanding. The main findings of this study were as follows: (1) In the Chinese population, the onset of STEMI exhibited a bimodal circadian rhythm pattern with a primary peak at 8:38 AM and a secondary peak at 12:55 PM. (2) For the onset of STEMI on every day of the week, the best-fit distribution is all shown as a bimodal curve. The onset time of the two peaks was delayed on Sunday, and the secondary peak became clearer. (3) The bimodal distribution pattern of STEMI onset persisted in subgroups of age, sex, and other traditional coronary risk factors. (4) There was no significant correlation between symptom onset time and MACCE.

### 4.1 Circadian rhythm of STEMI onset

We discovered a bimodal distribution of STEMI case density in the Chinese population, with an early peak of symptom onset at 8:38 AM, a subsequent spike at 12:55 PM, and a progressive decline in ischemia in the evening and at night. This finding was in line with previous studies that reported acute cardiovascular events had a circadian pattern, with symptoms most commonly occurring between 6:00 AM and 12:00 AM (2–6). Secondary peaks in the afternoon or evening have also been reported in minority studies (10). On the contrary, the investigation of Masiewicz and Sari et al. showed that the afternoon was the most common time for the circadian rhythm variant of myocardial infarction (18, 19). Some controversies remain regarding the peak distribution of the onset time of myocardial infarction. Most of the above results convert continuous-time variables into quadruple-categorical data, which could lead to information loss and thus reduce statistical power (20). Consequently, we remedied these shortcomings by assessing circadian rhythm variations in STEMI incidents among a large prospective myocardial infarction cohort using a more sensitive and robust statistical approach (i.e., maximum likelihood estimation fitting the von Mises distribution). In summary, our findings indicated a characteristic bimodal pattern of STEMI onset across 24 h, albeit with a less prominent and earlier peak in the afternoon.

The primary peak of STEMI onset may be correlated with changes in some physiological parameters. In the morning, sympathetic nerve activity, catecholamine and cortisol levels, blood pressure, heart rate, and coronary artery tone are all increased (7). Furthermore, there is an elevated risk of thrombosis due to increased platelet aggregation and adhesion, decreased plasma fibrinolytic activity (9), and decreased vascular endothelial function after morning rise (8). These physiological changes may lead to an increased likelihood of unstable plaque rupture and myocardial infarction in the morning. As confirmed by the OCT study, atherosclerotic plaque rupture is more frequent in the morning (21).

A novel finding of this study is the observation of a second peak in STEMI onset at 12:55 PM. Perhaps phenomenon correlates with the traditional Chinese eating habits. In China, the lunch is generally considered the largest meal of the day. Lipovetzky's study showed a significantly increased risk of acute coronary syndrome after intake of large meals (22). Furthermore, increased physical activity in the afternoon can also trigger a small degree of myocardial ischemia (23).

### 4.2 Heterogeneity of bimodal circadian rhythm distribution

The bimodal distribution pattern of STEMI onset was unaffected by pre-determined stratification variables, with only the minority of subgroups exhibiting slightly altered times of the second peak. A regular sleep-wake schedule may cause similar primary peak times on weekdays. The sudden increase in physical activity and mental stress from a rest day to a weekday encourages leukocyte aggregation in atherosclerosis, contributing to vascular inflammation and plaque instability and raising the risk of myocardial infarction (24), which explains the possible mechanism for the sharpest primary peaks on Monday and Tuesday. On Sunday, both peaks were delayed, with the primary peak late by close to 1.5 h, and the second peak occurred at night (22:30 PM) and was more apparent. Two factors may explain this phenomenon: Firstly, on work-free days, people wake up about one hour forty minutes later than on work days (25), whereas the peak of myocardial infarction incidence is mostly around one to two hours after the person wakes up (26). Secondly, weekend evenings are typically used for social activities and family get-together. During these times, people are prone to mood swings and overeating, which puts additional strain on their hearts (27). These findings may have potential implications for medical resource allocation to weekends, especially weekends night, when hospitals face shortages of doctors and interventional cardiologists during these times. Females may account for a significant portion of the second peak, according to a small sample study that included 522 STEMI patients (28). Nevertheless, our results agree with those of Xu's (3), indicating that male and female circadian rhythm variations are consistent. The results about age subgroups align with earlier data that indicated elderly AMI patients (>65 years) have nearly no late peak and a very pronounced early peak (29).

### 4.3 Timing of STEMI onset and clinical prognosis

The effect of the timing of STEMI onset on prognosis remains controversial. A recent study showed nocturnal onset was independently linked to an increased risk of long-term adverse cardiovascular events (12). However, the study by Sager et al. demonstrated that the timing of onset was not significantly associated with infarct size or 5-year all-cause mortality (13). Our large-sample, multi-center study found no association between the timing of STEMI onset and mortality or the incidence of MACCE, even after adjusting for cardiovascular risk factors and information related to coronary intervention. In the present study, cardiogenic shock was an independent predictor of 1-year MACE or mortality in patients with STEMI, supporting the previous studies that cardiogenic shock is the primary cause of poor prognosis after myocardial infarction (30, 31). Tokarek et al. found that the femoral approach was independently associated with higher periprocedural mortality compared with radial approach in patients with STEMI (32), along with our results.

In general, longer symptom-to-admission times were associated with larger myocardial infarct size (33) and worse prognosis (34). Previous studies have shown that the severity of coronary lesions and the door-to-balloon time balloon time were important factors affecting the prognosis of STEMI (35, 36). In addition, previous studies have also found that STEMI patients presenting during off-hours have longer door-to-balloon times and poorer clinical outcomes (37, 38). Although patients with onset time at 0:00–5:59 AM have longer prehospital delays, the time of STEMI onset usually did not significantly impact our treatment strategy, as door-to-balloon time, data related to PCI, and the medication at discharge were comparable among the four groups. Notably, onset-to-admission time and door-to-balloon time did not significantly affect the MACCE or mortality in our study. STEMI is an acute cardiovascular event, and timely medical intervention in the hospital may weaken the correlation between circadian rhythm and poor prognosis. This suggests the importance of raising awareness of STMEI symptoms and activating the medical system as early as possible. Notedly, further confirmation of these findings is necessary through a larger prospective study.

## 5 Limitations

The limitations of the present study are as follows. Firstly, this was a secondary analysis of multi-center research, with a few patients (7.5%) not having a clear time of onset of persistent chest pain. Secondly, the time of symptom onset was self-reported by the patients and dependent on subjective perceptions. Thirdly, this study did not consider the level of physical activity prior to symptom onset, as well as the patients' working hours and work status. Disruptions in physiological rhythms may affect our results. Finally, all patients were enrolled in China. So, our findings might not be generalizable to patients in other countries. Although the present study was based on a relatively large STEMI cohort, the sample size was still relatively low. Further prospective studies with larger sample sizes are warranted to investigate the circadian rhythm of myocardial infarction to confirm the generalizability of our results.

## 6 Conclusion

In the Chinese population, the onset of STEMI followed a specific circadian rhythm pattern with a bimodal distribution, including a clear primary peak at 8:38 AM and a less clear secondary peak at 12:55 PM. The timing of STEMI onset did not affect our treatment strategy and 1-year clinical outcomes.

## Data Availability

The original contributions presented in the study are included in the article/Supplementary Material, further inquiries can be directed to the corresponding author/s.
