# Supplementary material for: Circadian rhythm pattern of symptom onset in patients with ST-segment elevation myocardial infarction in the Chinese population
Source: Front Cardiovasc Med. 2024 Dec 10;11:1393390. doi: 10.3389/fcvm.2024.1393390 (PMC11666443; doi:10.3389/fcvm.2024.1393390)
Supplement: Supplementary file 1 [file Datasheet1.pdf]

Supplementary Material

1 Definition and diagnosis of coronary risk factors

Diabetes mellitus was diagnosed if a patient met 1 of the following criteria: documented history or self-reported clinician diabetes mellitus, taking hypoglycemic medicine, fasting glucose ≥126mg/dL, 2h plasma glucose level ≥200mg/dL, classic symptom with casual plasma glucose level ≥200mg/dL, or hemoglobin A1c (HbA1c) ≥6.5%[1]. Hypertension was diagnosed as systolic blood pressure ≥140mmHg or diastolic blood pressure ≥90mmHg or current use of anti-hypertensive agents. Dyslipidemia was defined as total cholesterol (TC) level ≥220mg/dL, triglycerides ≥150 mg/dL, low-density lipoprotein cholesterol (LDL-C) ≥140mg/dL, high-density lipoprotein cholesterol (HDL-C) ≤40mg/dL, or current use of agents for dyslipidemia[2]. Obesity was defined as body mass index (BMI) ≥30kg/m^2.

2 Definitions of major adverse cardiovascular and cerebrovascular events

All-cause death was defined as death from any cause occurring during follow-up. Recurrent myocardial infarction was defined as typical chest pain accompanied by a rise of more than two times the upper reference limit of troponins, development of new Q waves on the ECG, or both. Coronary revascularization was defined as unplanned percutaneous coronary intervention or coronary artery bypass grafting driven by ischemia or non-ischemia. Stroke could be either ischemic or hemorrhagic and was defined as a sudden onset of focal neurologic deficit caused by a vascular lesion in the brain that last for more than 24 h.

References

[1] American Diabetes A. Diagnosis and classification of diabetes mellitus [J]. Diabetes Care, 2010, 33 Suppl 1(Suppl 1): S62-9.

[2] Dai J, Xing L, Jia H, Zhu Y, Zhang S, Hu S, Lin L, Ma L, Liu H, Xu M, Ren X, Yu H, Li L, Zou Y, Zhang S, Mintz GS, Hou J, Yu B. In vivo predictors of plaque erosion in patients with ST-segment elevation myocardial infarction: a clinical, angiographical, and intravascular optical coherence tomography study [J]. Eur Heart J, 2018, 39(22): 2077-85.

[3] Levey AS, Stevens LA, Schmid CH, Zhang YL, Castro AF, 3rd, Feldman HI, Kusek JW, Eggers P, Van Lente F, Greene T, Coresh J, Ckd EPI. A new equation to estimate glomerular filtration rate [J]. Ann Intern Med, 2009, 150(9): 604-12.

Supplementary Figures

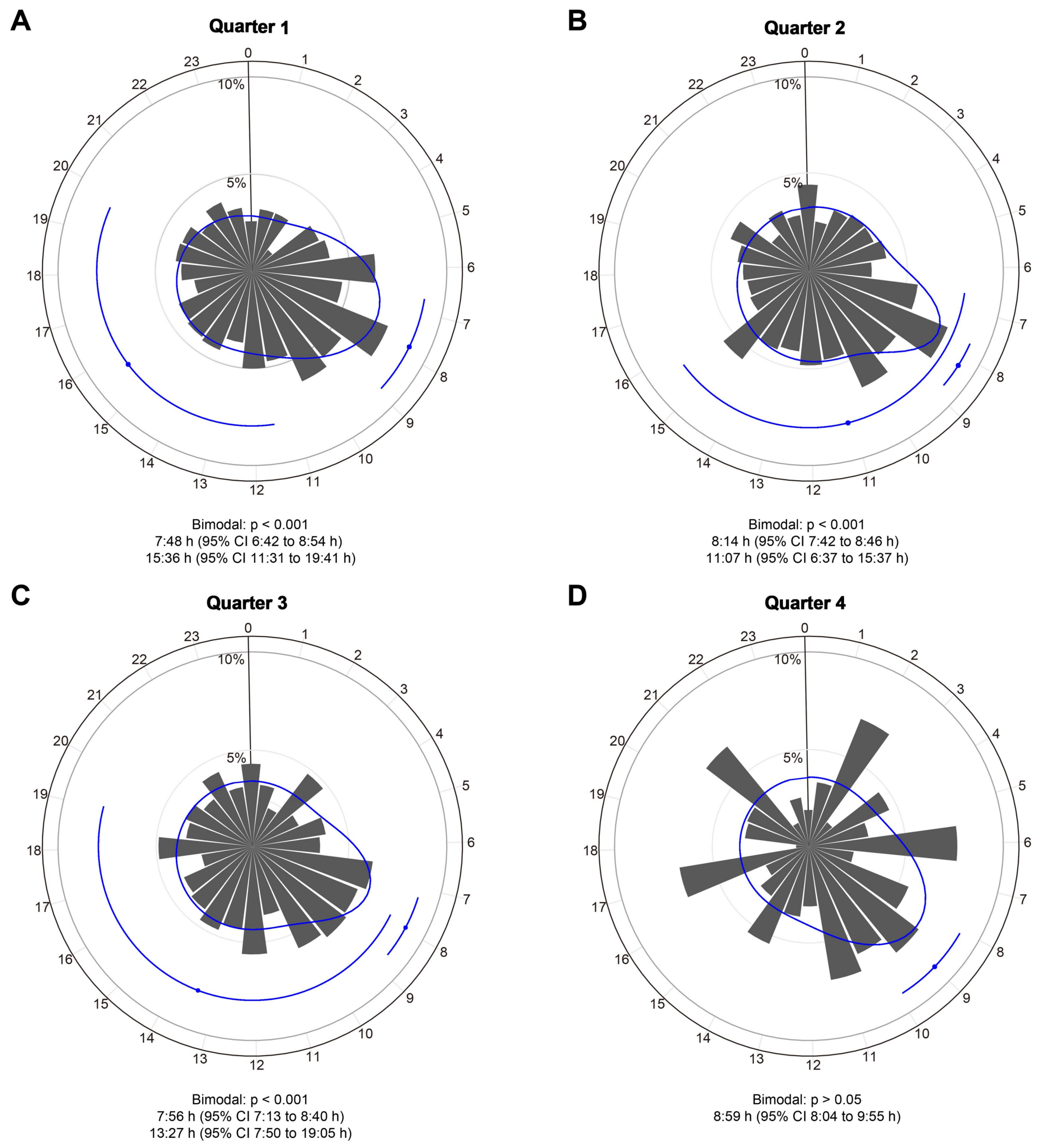

Supplementary Figure 1. Circadian rhythm pattern of STEMI onset based on the quarters of the year

Panel (A-D) shows circular plots fitting the von Mise distribution based on the quarters of the year. The estimated peak onset times and 95% CIs are shown below each circular plot. *p* values are used to assess whether the circadian rhythm pattern of STEMI onset was uniform, unimodal or bimodal. Quarter 1: January-March, Quarter 2: April-June, Quarter 3: July-September, and Quarter 4: October-December. CI, confidence interval; STEMI, ST-segment elevation myocardial infarction.

Supplementary Tables

Supplementary Table 1. Postoperative complications according to time of STEMI onset in patients with PCI.

	Group A (n=1,466)	Group B (n=2,409)	Group C (n=1,828)	Group D (n=1,516)	*P* value
Coronary artery perforation	0 (0.0)	0 (0.0)	2 (0.1)	1 (0.1)	0.277
Acute occlusion	2 (0.1)	0 (0.0)	1 (0.1)	0 (0.0)	0.181
No reflow	11 (0.7)	18 (0.7)	15 (0.8)	7 (0.5)	0.632
Pericardial tamponade	0 (0.0)	0 (0.0)	0 (0.0)	1 (0.1)	0.288
Air embolism	0 (0.0)	0 (0.0)	0 (0.0)	0 (0.0)	NA
Coronary artery dissection	1 (0.1)	1 (0.0)	1 (0.1)	2 (0.1)	0.756
Others	15 (1.0)	29 (1.2)	19 (1.0)	13 (0.9)	0.782

Data are presented as number (%). PCI, Percutaneous coronary intervention; STEMI, ST-segment elevation myocardial infarction.

Supplementary Table 2. Clinical events that occurred during hospitalization according to time of STEMI onset.

	Group A (n=1,591)	Group B (n=2,618)	Group C (n=1,957)	Group D (n=1,639)	*P* value
In-hospital death	24 (1.51)	40 (1.53)	27 (1.38)	21 (1.28)	0.911
In-hospital stroke, n (%)	1 (0.1)	1 (0.0)	2 (0.1)	1 (0.1)	0.927
In-hospital bleeding, n (%)	2 (0.1)	3 (0.1)	2 (0.1)	1 (0.1)	0.964
Intracranial bleeding	1 (0.1)	2 (0.1)	0 (0.0)	0 (0.0)	0.549
Gastrointestinal bleeding	1 (0.1)	1 (0.0)	0 (0.0)	0 (0.0)	0.691
Other bleeding	0 (0.0)	0 (0.0)	2 (0.1)	1 (0.1)	0.267

Data are presented as number (%). Abbreviation as shown in Supplemental Table 1.

Supplementary Table 3. Medications at discharge

	Group A (n=1,591)	Group B (n=2,618)	Group C (n=1,957)	Group D (n=1,639)	*P* value
Aspirin	1494 (93.9)	2486 (95.0)	1851 (94.6)	1547 (94.4)	0.530
Clopidogrel/ticagrelor	1495 (94.0)	2491 (95.1)	1859 (95.0)	1558 (95.1)	0.355
Statins	1489 (93.6)	2470 (94.3)	1844 (94.2)	1550 (94.6)	0.658
β-blockers	1114 (70.0)	1841 (70.3)	1366 (69.8)	1191 (72.7)	0.228
ACEI/ARB	905 (56.9)	1470 (56.1)	1129 (57.7)	963 (58.8)	0.383

Values are presented as n (%). ACEI = angiotensin-converting enzyme inhibitor; ARB = angiotensin receptor blocker.

Supplementary Table 4. Angiographic findings and procedural characteristics in patients with PCI.

	Group A (n=1,466)	Group B (n=2,409)	Group C (n=1,828)	Group D (n=1,516)	*P* value
**Angiographic findings**					
Lesion vessel					0.052
LAD	730 (49.8)	1151 (47.8)	904 (49.5)	766 (50.5)	
LCX	163 (11.1)	251 (10.4)	188 (10.3)	191 (12.6)	
RCA	548 (37.4)	981 (40.7)	710 (38.8)	549 (36.2)	
LM	12 (0.8)	10 (0.4)	12 (0.7)	5 (0.3)	
Others	13 (0.9)	16 (0.7)	14 (0.8)	5 (0.3)	
TIMI flow grade 0/1	970 (66.2)	1522 (63.2)	1190 (65.1)	1017 (67.1)	0.064
Diameter stenosis according to visual assessment, %	96.5 ± 8.3	96.2 ± 8.3	96.7 ± 7.4	96.7 ± 7.3	0.195
**Door-to-balloon time, min**	46.0 (35.0-63.0)	45.0 (34.0-62.0)	44.0 (33.0-60.0)	47.0 (35.0-61.0)	0.064
**Procedural characteristics**					
Surgical approach					0.229
Radial approach	1285 (87.7)	2132 (88.5)	1627 (89.0)	1347 (88.9)	
Femoral approach	160 (10.9)	231 (9.6)	163 (8.9)	150 (9.9)	
Brachial approach	21 (1.4)	46 (1.9)	38 (2.1)	19 (1.3)	
Stent implantation	1180 (80.5)	1964 (81.5)	1506 (82.4)	1231 (81.2)	0.514
Number of stents, n	1.3 ± 0.5	1.3 ± 0.5	1.3 ± 0.6	1.3 ± 0.5	0.244
Stent length, mm	28.3 ± 10.5	28.4 ± 9.7	28.9 ± 11.5	28.0 ± 9.8	0.099
Stent diameter, mm	3.1 ± 0.4	3.1 ± 0.4	3.1 ± 0.4	3.1 ± 0.5	0.448
Pre-dilation	1166 (79.5)	1906 (79.1)	1503 (82.2)	1210 (79.8)	0.052
Post-dilation	970 (66.2)	1544 (64.1)	1208 (66.1)	957 (63.1)	0.177

Values are presented as n (%), mean ± SD, or median (IQR). LAD = left anterior descending artery; LCX = left circumflex artery; LM = left main coronary artery; RCA = right coronary artery; TIMI = initial thrombolysis in myocardial infarction.

Supplementary Table 5. Univariate and multivariate cox regression analysis for MACCE or all-cause death.

	Univariate analysis		Multivariate analysis	
	HR (95%CI)	*P*-value	HR (95%CI)	*P*-value
**MACCE**				
The timing of STEMI onset				
Group B	1 (Reference)		1 (Reference)	
Group A	1.04 (0.79-1.37)	0.791	0.82 (0.54-1.26)	0.370
Group C	1.10 (0.85-1.43)	0.461	1.03 (0.70-1.51)	0.900
Group D	1.08 (0.82-1.41)	0.603	1.13 (0.76-1.67)	0.544
Age	1.04 (1.03-1.05)	<0.001	1.03 (1.02-1.05)	<0.001
Female	1.13 (0.91-1.40)	0.288		
BMI	0.95 (0.93-0.98)	0.002	0.99 (0.95-1.03)	0.600
Smokers	0.81 (0.67-0.99)	0.045	1.05 (0.77-1.42)	0.772
Drinkers	0.85 (0.66-1.10)	0.219		
Dyslipidemia	0.87 (0.48-1.59)	0.656		
Hypertension	1.24 (1.02-1.50)	0.035	0.97 (0.72-1.32)	0.865
Diabetes	1.32 (1.06-1.65)	0.012	0.87 (0.58-1.31)	0.507
Previous PCI	1.34 (0.91-1.98)	0.144		
Previous stroke	1.89 (1.49-2.40)	<0.001	1.29 (0.89-1.87)	0.182
Cardiogenic shock at admission	8.11 (2.02-32.59)	0.003	129.78 (22.45-613.66)	<0.001
Hs-CRP	1.01 (1.00-1.01)	<0.001	1.00 (0.99-1.01)	0.756
NT-pro BNP	1.00 (1.00-1.00)		1.00 (1.00-1.00)	<0.001
CK-MB	1.00 (0.99-1.00)	0.660		
cTnI	1.00 (1.00-1.00)	0.983		
FBG	1.05 (1.03-1.08)	<0.001	1.03 (0.99-1.09)	0.170
HbA1c	1.00 (0.98-1.02)	0.979		
Onset-to-admission time	1.00 (1.00-1.00)	0.861		
Door-to-balloon time	0.99 (0.99-1.00)	0.286		
Aspirin	1.19 (0.74-1.91)	0.477		
Clopidogrel/ticagrelor	1.45 (0.85-2.47)	0.174		
Statins	1.43 (0.87-2.36)	0.160		
β-blockers	1.35 (1.07-1.70)	0.012	1.34 (0.91-1.99)	0.139
ACEI/ARB	1.33 (1.09-1.64)	0.006	0.86 (0.62-1.20)	0.376
TIMI flow grade 0/1	0.96 (0.77-1.19)	0.709		
Diameter stenosis according to visual assessment	1.01 (0.99-1.02)	0.340		
Surgical approach				
Radial approach	1 (Reference)			
Femoral approach	1.31 (0.95-1.81)	0.104		
Brachial approach	1.24 (0.59-2.62)	0.574		
**All-cause death**				
The timing of STEMI onset				

Group B	1 (Reference)		1 (Reference)	
Group A	1.30 (0.86-1.95)	0.215	1.24 (0.63-2.45)	0.532
Group C	1.42 (0.97-2.07)	0.071	1.37 (0.72-2.61)	0.333
Group D	1.14 (0.75-1.74)	0.547	1.31 (0.67-2.57)	0.432
Age	1.08 (1.06-1.09)	<0.001	1.04 (1.01-1.07)	0.002
Female	1.65 (1.23-2.23)	0.001	0.76 (0.43-1.34)	0.341
BMI	0.89 (0.85-0.93)	<0.001	0.98 (0.91-1.06)	0.617
Smokers	0.64 (0.47-0.87)	0.004	0.82 (0.48-1.40)	0.471
Drinkers	0.61 (0.40-0.94)	0.023	1.02 (0.54-1.95)	0.951
Dyslipidemia	0.33 (0.08-1.34)	0.121		
Hypertension	1.31 (0.98-1.75)	0.068	0.88 (0.53-1.44)	0.599
Diabetes	1.18 (0.85-1.64)	0.333		
Previous PCI	0.82 (0.40-1.66)	0.578		
Previous stroke	2.23 (1.59-3.12)	<0.001	1.66 (0.94-2.93)	0.080
Cardiogenic shock at admission	8.00 (1.12-57.09)	0.038	57.99 (5.66-594.12)	0.001
Hs-CRP	1.01 (1.01-1.02)	<0.001	1.00 (0.99-1.01)	0.700
NT-pro BNP	1.00 (1.00-1.00)	<0.001	1.00 (0.99-1.00)	0.139
CK-MB	1.00 (0.99-1.00)	0.883		
cTnI	1.00 (0.99-1.00)	0.884		
FBG	1.05 (1.01-1.09)	0.021	1.03 (0.96-1.10)	0.397
HbA1c	0.99 (0.96-1.04)	0.895		
Onset-to-admission time	1.00 (1.00-1.00)	0.735		
Door-to-balloon time	1.00 (0.99-1.00)	0.667		
Aspirin	0.99 (0.53-1.88)	0.980		
Clopidogrel/ticagrelor	1.35 (0.64-2.88)	0.432		
Statins	1.33 (0.66-2.71)	0.428		
β-blockers	0.94 (0.68-1.28)	0.673		
ACEI/ARB	0.92 (0.69-1.23)	0.567		
TIMI flow grade 0/1	1.21 (0.85-1.72)	0.280		
Diameter stenosis according to visual assessment	1.03 (0.99-1.06)	0.060	1.03 (0.98-1.09)	0.189
Surgical approach				
Radial approach	1 (Reference)		1 (Reference)	
Femoral approach	2.38 (1.58-3.58)	<0.001	1.98 (1.13-3.49)	0.018
Brachial approach	1.83 (0.67-4.95)	0.236	0.72 (0.10-5.25)	0.745

Values are presented as HR (95%CI). All the variables that showed *P* values <0.1 in the univariate model and the timing of STEMI onset were simultaneously entered into the multivariate model. The timing of STEMI onset using the Group B as the reference, due to the lower rate of incident event. MACCE was defined as the composite of all-cause death, recurrent myocardial infarction, revascularization, and stroke. BMI, Body mass index; CI, confidence interval; CK-MB, Creatine kinase-myocardial band; cTnI, cardiac troponin I; FBG, fasting blood-glucose; HbA1c, Hemoglobin A1C; Hs-CRP, High sensitivity C reactive protein; HR, hazards ratio; MACCE, major adverse

cardiovascular and cerebrovascular events; NT-pro BNP, N-terminal pro–brain natriuretic peptide. Other abbreviations as shown in Supplemental Tables 1, 2 and 3.

Supplementary Table 6. Clinical outcomes at one year according to time of STEMI onset in patients admitted before 2019.

	Group A (n=553)	Group B (n=867)	Group C (n=675)	Group D (n=609)	*P* value
MACCE, n (%)	25 (4.7)	51 (6.0)	45 (6.7)	44 (7.3)	0.302
All-cause death, n (%)	7 (1.3)	15 (1.8)	19 (2.8)	16 (2.6)	0.199
Recurrent MI, n (%)	0 (0.0)	9 (1.1)	8 (1.2)	4 (0.7)	0.089
Revascularization, n (%)	13 (2.5)	29 (3.4)	18 (2.7)	20 (3.3)	0.708
Stroke, n (%)	5 (0.9)	5 (0.6)	4 (0.6)	9 (1.5)	0.249
Bleeding	3 (0.6)	9 (1.1)	7 (1.0)	8 (1.3)	0.647
Intracranial hemorrhage	1 (0.2)	1 (0.1)	1 (0.1)	2 (0.3)	0.874
Gastrointestinal bleeding	2 (0.4)	6 (0.7)	1 (0.1)	4 (0.7)	0.382
Other bleeding	0 (0.0)	2 (0.2)	5 (0.7)	2 (0.3)	0.165

Data are presented as number (%). MACCE was defined as the composite of all-cause death, recurrent myocardial infarction, revascularization, and stroke. MACCE, major adverse cardiovascular and cerebrovascular events; MI, myocardial infarction; STEMI, ST-segment elevation myocardial infarction.
